# Understanding the Impact of Aberrant Splicing in Coagulation Factor V Deficiency

**DOI:** 10.3390/ijms20040910

**Published:** 2019-02-20

**Authors:** Elvezia Maria Paraboschi, Marzia Menegatti, Flora Peyvandi, Stefano Duga, Rosanna Asselta

**Affiliations:** 1 Department of Biomedical Sciences, Humanitas University, Via Rita Levi Montalcini 4, Pieve Emanuele, 20090 Milan, Italy; elvezia_maria.paraboschi@hunimed.eu (E.M.P.); stefano.duga@hunimed.eu (S.D.); 2 Humanitas Clinical and Research Center, IRCCS, Via Manzoni 56, Rozzano, 20089 Milan, Italy; 3 Angelo Bianchi Bonomi Haemophilia and Thrombosis Centre, Fondazione IRCCS Ca’ Granda Ospedale Maggiore Policlinico, and Luigi Villa Foundation, 20100 Milan, Italy; marzia.menegatti@guest.unimi.it (M.M.); flora.peyvandi@unimi.it (F.P.); 4 Department of Pathophysiology and Transplantation, Università degli Studi di Milano, 20100 Milan, Italy

**Keywords:** coagulation factor V, factor V deficiency, splicing mutations, minigene expression experiments, mutational spectrum

## Abstract

Rare inherited coagulation disorders (RICDs) are congenital deficiencies of the plasma proteins that are involved in blood coagulation, which generally lead to lifelong bleeding manifestations. These diseases are generally qualitative and/or quantitative defects that are associated with monoallelic or biallelic mutations in the relevant gene. Among RICDs, factor V (FV) deficiency is one of the least characterized at the molecular level. Here, we investigated four unrelated patients with reduced plasma FV levels (three severe, one mild), which were associated with a moderately severe bleeding tendency. Sequence analysis of the FV gene identified seven different variants, five hitherto unknown (p.D1669G, c.5789-11C>A, c.5789-12C>A, c.5789-5T>G, and c.6528G>C), and two previously reported (c.158+1G>A and c.5789G>A). The possible pathogenic role of the newly identified missense variant was studied by in silico approaches. The remaining six genetic defects (all putative splicing mutations) were investigated for their possible effects on pre-mRNA splicing by transient transfection experiments in HeLa cells with plasmids expressing appropriate hybrid minigenes. The preparation of minigene constructs was instrumental to demonstrate that the two adjacent variants c.5789-11C>A and c.5789-12C>A are indeed present in cis in the analyzed FV-deficient patient (thus leading to the c.5789-11_12CC>AA mutation). Ex vivo experiments demonstrated that each variant causes either a skipping of the relevant exon or the activation of cryptic splice sites (exonic or intronic), eventually leading to the introduction of a premature termination codon.

## 1. Introduction

Factor V (FV), which is also known as proaccelerin or labile factor, is a 330-kDa multi-domain (A1–A2–B–A3–C1–C2) procofactor of the coagulation cascade showing no intrinsic coagulant activity until its conversion to activated FV (FVa) [1,2,3]. FV activation occurs by a limited proteolysis, which is exerted either by thrombin or activated factor X (FXa) at specific arginine residues, i.e., p.Arg709, p.Arg1018, and p.Arg1545; this results in the removal of the large B domain [1,2,3]. Once activated, FVa, together with FXa, participates in the formation of the prothrombinase complex, which is responsible for the generation of thrombin [1,2,3]. FV also shows an anticoagulant activity, by acting (together with protein S) in the activated protein C-mediated inactivation of activated factor VIII [4].

FV deficiency (Online Mendelian Inheritance in Man #227400) is an inherited coagulation disorder that was originally described by Owren in 1947 as a hemorrhagic diathesis due to the absence of a previously unknown coagulation factor [5]. A few years later, the modality of transmission (autosomal recessive) became clear [6], whereas the isolation and molecular characterization of homogeneous FV was achieved only in 1979 [7]. FV deficiency is rare, with an estimated prevalence of one per million in the general population, but in countries where consanguineous marriages are frequent, the disorder can occur up to 10 times more frequently [8,9]. The disease can be classified as either type-I deficiency—which is characterized by FV antigen levels that can be mildly reduced, low, or even unmeasurable (quantitative defect)—or type-II deficiency, showing normal or slightly-decreased antigen levels associated with reduced coagulant activity (qualitative defect) [10]. Altered levels of the coagulation factor are responsible for clinical manifestations going from life-threatening bleeding episodes (mostly in the gastrointestinal tract and the central nervous system) to less severe symptoms, including epistaxis, menorrhagia, easy bruising, hemarthroses, and hematomas [10,11].

FV quantitative defects are associated with monoallelic and biallelic mutations in the FV gene (*F5*), which consists of 25 exons, covers a genomic region of 74.6 kb, and is located on chromosome 1q24.2 (Human genome assembly GRCh37/hg19; UCSC Genome Browser, http://genome.ucsc.edu/) (access on 20 December 2018). According to the publicly available version of the Human Gene Mutation Database (HGMD, http://www.hgmd.cf.ac.uk/ac/index.php) (access on 20 December 2018), a total of 134 genetic defects have been described as associated with FV deficiency so far; among them, 14 mutations (10.4%) affecting splicing have been identified, even though these have not been functionally investigated in all of the cases [12,13,14,15,16,17,18,19,20,21,22,23,24].

In this work, we report the full molecular and functional characterization of four patients affected by FV deficiency. The study was carried out through an initial sequencing of the *F5* coding sequence, and by a subsequent combination of in silico analyses and ex vivo expression experiments. In addition, a detailed review of the literature with a specific focus on *F5* splicing defects, as well as data mining in publicly available mutation/variation databases, revealed that a great proportion of such variants are indeed due to misinterpreted missense/synonymous substitutions falling in the exonic regions of splicing sites.

## 2. Results

### 2.1. Clinical Details of the Patients

Four unrelated patients (P1–P4) with congenital FV deficiency were included in this study. They were enrolled among consecutive patients referring to our collaborating centers on the basis of a coagulatory screening revealing a FV coagulant activity (FV:C) < 5% in three cases, and FV:C = 56% in the remaining one. The main clinical features of these patients are summarized in Table 1.

### 2.2. Identification of Molecular Defects

The mutational screening was performed by conventional Sanger sequencing on PCR-amplified genomic fragments covering the whole *F5* coding region, splice sites, and ~300 bp of the promoter region.

We disclosed a total of seven different variants, two of which (c.158+1G>A and c.5789G>A/p.G1902D) were recurring in two subjects (Table 2) and previously reported in an Italian patient with severe FV deficiency [24]. These two variants were indeed described as part of a unique complex allele, but no functional studies were undertaken to dissect their actual contribution to the disease. Of note, the c.5789G>A/p.G1902D variant involves the first nucleotide of exon 20, hence possibly impacting on *F5* splicing.

As for the remaining five novel variants, we identified one putative missense substitution, three splicing defects (two of which are in adjacent positions in intron 19), and an additional missense variant (c.6528G>C/p.K2148N) falling in the last nucleotide of exon 24, thus again potentially influencing the *F5* pre-mRNA splicing process (Table 2).

The identified mutations are either absent or reported in the Genome Aggregation database (GnomAD; http://gnomad.broadinstitute.org/) at an extremely low frequency in the general population (highest frequency was 1.42 × 10^−5^ for the c.5789G>A/p.G1902D variant, in which this allele was reported four times over a total of 281,772 alleles) (Table 2).

### 2.3. In Silico Analyses of the Identified Variants

All of the identified variants were submitted to computer-assisted analyses in order to predict their possible impact either on the *F5* pre-mRNA splicing, or on the FV protein structure. In particular, we accomplished splice-site predictions using four online tools on the six identified putative splicing variants; in addition, we used five algorithms for estimating the disruptive potential of the three missense substitutions (the “genuine” p.D1669G missense variant, plus the two “elusive” variants, c.5789G>A/p.G1902D and c.6528G>C/p.K2148N, both potentially interfering with the splicing process) (Table 2). A summary of the in silico prediction analyses is reported in Table 3.

As for the putative splicing defects, the prediction tools consistently recognized a significant difference between the wild-type and the mutant sequence only for the c.158+1G>A mutation, as well as for the two c.5789G>A/p.G1902D and c.6528G>C/p.K2148N variants, thus supporting their possible impact on mRNA splicing.

As for putative missense mutations, at least four (out of five) prediction programs consistently attributed a disruptive potential to the p.D1669G and c.6528G>C/p.K2148N variants. Conversely, the c.5789G>A/p.G1902D substitution was considered as not damaging by all five algorithms.

**Figure 1:**
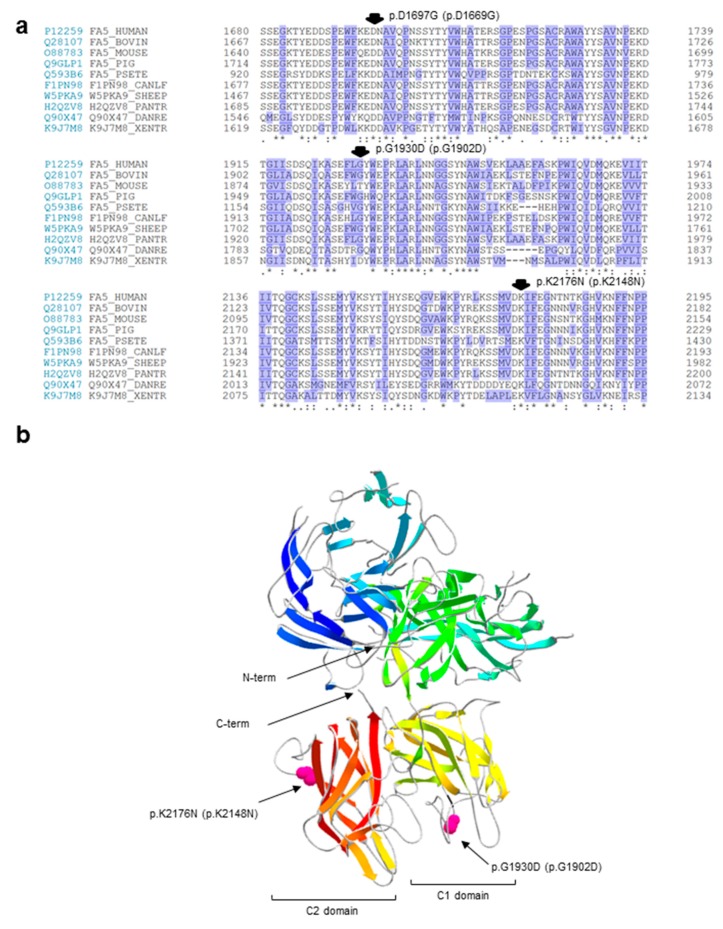
In silico charaterization of the putative missense mutations. (**a**) Multiple alignments of regions of coagulation FV that are 50 amino acids long from several vertebrates harboring the three identified missense variants. Protein sequences were obtained from the UniProt database (https://www.uniprot.org/) under the accession numbers P12259 (human, *Homo sapiens*), Q28107 (bovin, *Bos taurus*), O88783 (mouse, *Mus musculus*), Q9GLP1 (pig, *Sus scrofa*), Q593B6 (psete, *Pseudonaja textilis*), F1PN98 (canlf, *Canis lupus familiaris*), W5PKA9 (sheep, *Ovis aries*), H2QZV8 (pantr, *Pan troglodytes*), Q90X47 (danre, *Danio rerio*), and K9J7M8 (xentr, *Xenopus tropicalis*). Residues are numbered, including the signal peptide. Conserved amino acids are indicated by asterisks (perfect identity among species), colons (high conservation), and dots (low conservation). Hydrophobic residues are shaded in violet. The three identified missense variants are indicated by arrows and according to their native and mature protein numbering. (**b**) Ribbon diagrams of the secondary and tertiary structures of the bovine inactive FVa (FVai) are shown. The positions of two out of three putative missense mutations are indicated in pink (the region harboring the p.D1669G variant is not included in the FVai structure). Mutation numbering refers to the highly homologous human structure. The color code indicates the different FVai domains (shades of yellow and red point to the C1 and C2 domains, respectively). The amino-termini (N) and carboxy-termini (C) of the entire structure are also indicated.

To give further insights on the possible pathogenic role of the amino-acid substitutions, we also carried out: (i) a sequence conservation analysis (Figure 1a)*,* by performing multiple alignments of coagulation FV sequences from several vertebrates in the regions harboring the identified variants; and (ii) a molecular modeling analysis (Figure 1b), using, as a template, the atomic coordinates of the structure of the activated protein-C inactivated bovine FVa (FVai), i.e., the sole available in the databases reporting the reconstruction of a complete molecular model for FV [25]. The first analysis evidenced that the p.D1669G variant is the only non-conservative amino acid substitution, involving a perfectly conserved residue; the mutation would introduce a small nonpolar amino acid (glycine) in a highly polar and conserved context (the consensus sequence of the region for the analyzed species is K/Q–E/Q–D–N/D, in which the third amino acid of this stretch is the one involved in the identified mutation; Figure 1a). These results, together with the above-mentioned outputs of prediction programs, strongly support the possible pathogenic role of the p.D1669G variant. Unfortunately, it was not possible to further investigate this variant by inspecting its position within the FVai three-dimensional structure, because this model lacks the parts of the FV molecule that are lost during the activation/inactivation processes. Concerning instead the c.5789G>A/p.G1902D and c.6528G>C/p.K2148N variants, both involve a residue that is part of a connecting unstructured loop, which is exposed on the protein surface (of the C1 and C2 domain, respectively; Figure 1b). The predicted substitutions would cause a change in the electrostatic signature of the relevant region, although the introduction of a charged/polar amino acid in a region exposed to the solvent could be in theory well tolerated.

### 2.4. Molecular Characterization of Splicing Mutations

**Figure 2:**
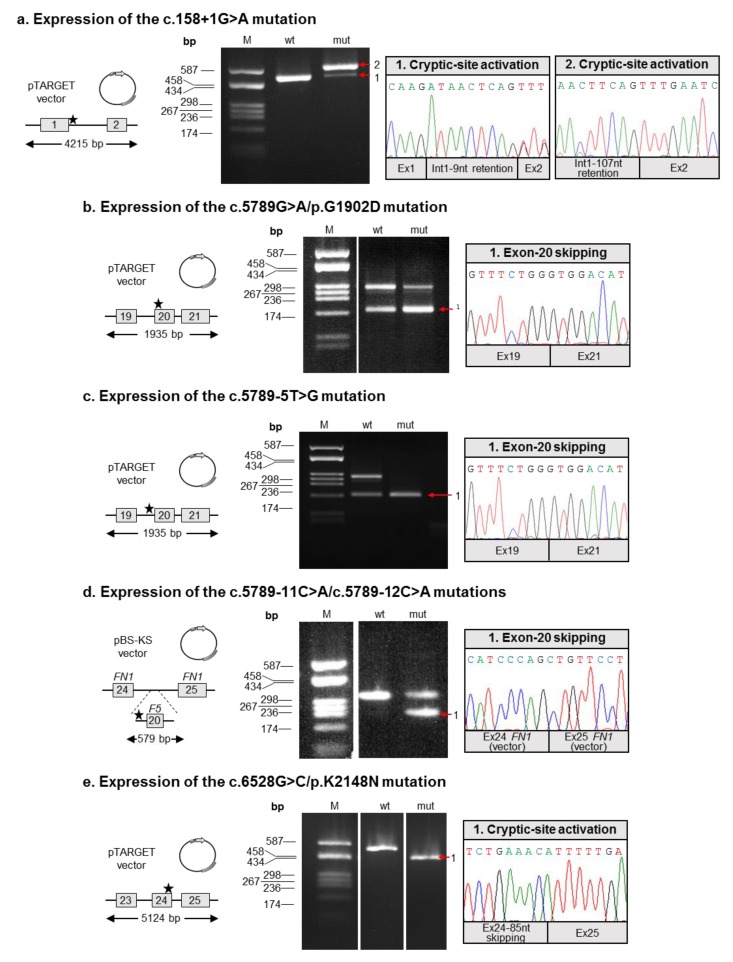
Functional characterization of the identified splicing mutations. (**a**–**e**) In each subgraph, the molecular characterization of a putative splicing mutation is reported (the name is indicated above the relevant panel). Left panels: Schematic representation of the *F5* regions cloned into expression vectors (either pTARGET or the pBS-KS chimeric construct). Exons are numbered and indicated by boxes, introns are represented by lines; exons and introns are not to scale. The approximate position of the mutation is indicated by a star. Middle panels: RT-PCR products obtained from the RNA of cells transfected with the minigene constructs, separated on an agarose gel. M: molecular weight marker (pUC9/HaeIII); wt: RT-PCR product derived from transfected HeLa cells expressing the wild-type minigene; mut: RT-PCR product derived from transfected HeLa cells expressing the mutant construct. Right panels: Electropherograms showing the sequences of aberrant splicing events resulting from the analyzed mutations.

Since in silico analyses were partially inconclusive, we decided to study the possible effects of all six putative splicing defects on *F5* splicing by an ex vivo approach. To this end, appropriate *F5* minigene constructs were generated, either in the wild-type form, or containing the relevant genetic defect (for details, see Materials and Methods; Figure 2). As a first result, this cloning strategy allowed us to verify that the c.5789-11C>A and c.5789-12C>A mutations are actually in cis, and were hence further characterized together.

All of the minigene constructs were transiently transfected into HeLa cells, and *F5* transcripts were examined by appropriate RT-PCR assays, followed by the sequencing of all the amplified products. This approach allowed us to demonstrate that all the investigated variants are indeed “true” splicing defects, including the “apparent” missense c.5789G>A/p.G1902D and c.6528G>C/p.K2148N substitutions.

In particular, three mutations (c.5789G>A/p.G1902D, c.5789-5T>G, and c.5789-11C>A/c.5789-12C>A) were associated with the inactivation of the physiologic splice site, thus resulting in the skipping of the corresponding exon (which was exon 20 in all cases). The skipping was total in the case of the c.5789-5T>G mutation (Figure 2c), whereas a detectable amount of residual wild-type splicing was associated with the c.5789G>A/p.G1902D and c.5789-11C>A/c.5789-12C>A mutations (Figure 2b,d).

As for the c.158+1G>A defect, the mutation was responsible for the disruption of the exon 1 donor splice site, with the concomitant activation of two cryptic splice sites (respectively located 9 and 107 nucleotides downstream of the physiologic one). The activation of these intronic splice sites was associated with the production of two aberrant transcripts, which were both characterized by the presence of a longer exon 1 (due to the retention of nine or 107 intronic nucleotides; see Figure 2a).

Regarding the c.6528G>C/p.K2148N mutation, this substitution was demonstrated to inactivate the physiologic donor splice site of exon 24, with the simultaneous activation of an upstream cryptic site, which was located within exon 24. This event was associated with the expression of a shorter *F5* transcript, which was characterized by a partial skipping (85 nucleotides) of exon 24 (Figure 2e).

The identified aberrant transcripts are predicted to determine the introduction of a premature termination codon (PTC), and hence trigger the mechanism of the nonsense-mediated mRNA decay. This process specifically recognizes PTC-containing transcripts, thus targeting them to degradation [26], and was previously shown to be active in the control of the post-transcriptional regulation of *F5* [18]. The only exception is represented by the transcript characterized by the in-frame retention of nine nucleotides of intron 1 (due to the mutation c.158+1G> A; Figure 2a), which however seems to be produced at extremely low levels.

## 3. Discussion

In this work, we investigated four patients—three Italians and one from Iran—who were affected by mild or severe FV deficiency. We identified a total of seven different and potentially disease-causing variants; however, understanding which among these defects were the actual mutations causing the deficiency was the most challenging task. Indeed, we experienced some unusual occurrences:

(1) We found a complex allele composed of a putative splicing defect (c.158+1G>A) and a missense variant (c.5789G>A/p.G1902D) in two unrelated subjects (P1 and P2). This allele was already described in the literature, but none of the putative mutations were functionally investigated [24];
(2) We identified two missense variants, one involving the first nucleotide (the above-mentioned c.5789G>A/p.G1902D) and the other involving the last nucleotide (c.6528G>C/p.K2148N) of the corresponding exon, hence both potentially interfering with the pre-mRNA splicing;
(3) We disclosed two adjacent nucleotide substitutions in the heterozygous state, in theory contributing to FV deficiency either in cis or in trans.

In order to clarify the actual contribution of all the identified putative splicing defects, it was hence necessary to tackle their in-depth functional characterization. The molecular study was based on expression experiments of ad-hoc minigene constructs, also to overcome the impossibility of having access to suitable specimens from the patients. With our ex-vivo approach, we were able to demonstrate that:

(1) Both mutations described by Bafunno et al. [24] can be regarded as splicing mutations. Since these two mutations are present in cis on the same allele, it is plausible that their combined effect can be considered a complete loss of function. It is worth noting that should a recombination event involve this allele, it would be responsible for the spreading of two different mutations: one severe (c.158+1G>A), and the other characterized by a milder effect, as we demonstrated that the c.5789G>A defect is associated with a certain degree of wild-type splicing (Figure 2);
(2) Similar to the c.5789G>A/p.G1902D mutation, the c.6528G>C/p.K2148N defect should also be regarded as a splicing rather than a missense mutation;
(3) The two adjacent mutations, c.5789-11C>A and c.5789-12C>A, are present in cis on the same allele, both possibly contributing to the splicing defect. Hence, we propose the c.5789-11_12CC>AA name for this mutation. Indeed, even though the two variants are reported as single alleles in the GnomAD database, inspection of the relevant sequencing reads (accessible from the same website) confirmed the phase of the two variants.

**Figure 3:**
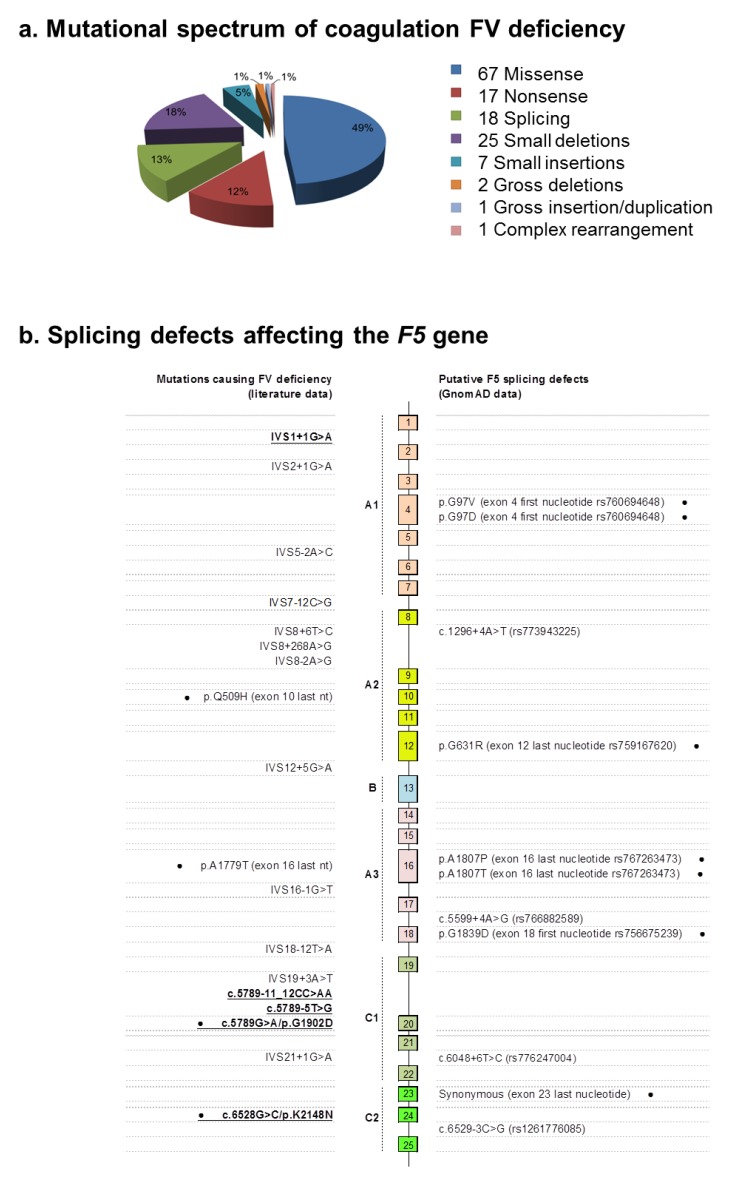
Mutational spectrum of FV deficiency. (**a**) The pie chart shows the distribution of the different types of mutations identified in the *F5* gene. Data were retrieved from the Human Gene Mutation Database (HGMD) database (public version) with some minor amendments: (i) two of the listed splicing defects are erroneously reported by the HGMD, and were not included in the figure; (ii) the missense mutation reported by Bafunno et al. [24] has been counted among the splicing defects. The chart also includes the four mutations identified in this work. (**b**) The mutational spectrum of the *F5* gene exclusively refers to the splicing defects. All of the known causative mutations for FV deficiency are projected on the exon–intron structure of *F5* on the left side of the scheme (data retrieved from the literature), whereas the putative splicing defects extracted from the GnomAD database are reported on the right (with the RefSeq indication, if available). Exons are represented by boxes; introns are represented by lines (not to scale). The FV domain structure is also indicated. Missense mutations affecting the first/last nucleotide of the exon are indicated by a black dot beside the name; the mutations functionally characterized in this work are bolded and underlined.

Altogether, our data allowed the identification of a total of four novel mutations (one missense, three splicings), bringing the total number of genetic defects that have been reported to cause mild or severe forms of FV deficiency to 138 (HGMD; database accessed 20 December 2018). Their frequency distribution according to mutation types is depicted in Figure 3a; in this figure, we also reported on and projected the exon–intron structure of the gene, including all of the known splicing mutations causing FV deficiency (a total of 18 genetic defects).

In the light of what we experienced in the present work, it clearly emerges that in the absence of experimental validations, the interpretation of the sequence variants found in genetic screenings still represents a great challenge for the diagnosis of inherited diseases, especially in the case of non-obvious mutations (e.g., missense variants and splicing mutations lying outside the ultra-conserved intronic dinucleotide of the splice sites). Actually, at least two additional missense substitutions causing FV deficiency were revealed to be splicing defects: (i) the p.Gln509His mutation, which is an apparent missense activating a cryptic donor splice site [14]; and (ii) the p.Ala1779Thr substitution, which interferes with intron 16 splicing [12]. Hence, four out of 18 splicing defects causing FV deficiency (22%) have been or could have been misinterpreted as missense mutations.

To have a global picture of the burden of the potentially deleterious splicing defects characterizing the *F5* gene, we performed a systematic search in exome/genome data from ~140,000 individuals belonging to the GnomAD database. Figure 3b reports all of the identified splicing variants (mapping at intronic positions −3/−1 and +3/+6) together with all the missense defects or synonymous variants falling in the first/last nucleotide of an exon, thus possibly interfering with the splicing process. In this search, we only considered high-quality variants, with minor allele frequencies <0.0001, and showing an ADA score >0.7 in prediction analyses (i.e., a score denoting potentially disruptive mutations; see the legend in Table 3). These stringent criteria, which already have been adopted in the literature for prevalence calculations of RICDs [27,28], allowed the identification of 11 putative splicing defects, including the already described p.Ala1807Thr mutation; however, no functional studies were performed to discern between the possible impact of this mutation either on the protein or on the transcript splicing [20]. Indeed, our in silico analysis indicates that a great proportion of splicing defects (54.5%) could be due to misinterpreted missense/synonymous variants. However, these data should be interpreted with caution, due to the lack of a proper functional characterization.

Besides the evident benefits from the diagnostic point of view, knowledge on the actual molecular mechanism underlying FV deficiency—and more in general, RICDs—may have further consequences on their management and therapy. In fact, considering that splicing defects often lead to the introduction of PTCs, the precise discernment between true null alleles and mutations that lead to single amino-acid substitutions could be of help for predicting (and managing) the development of inhibitors [10,29]. In addition, the knowledge of the actual splicing defect underlying the coagulation deficiency is the fundamental prerequisite for next-generation RNA-based therapeutic approaches [30]. These are considered possible new frontiers in the treatment of RICDs, since they would allow the restoration of the altered transcript, overcoming both the drawbacks that are typical of conventional gene therapy (e.g., tissue specificity, correct regulation of expression, the need to deliver the transgene), and possibly those deriving from the replacement therapy [30]. A final consideration pertains to an old “open question” regarding the characterization of RICDs, i.e., the lack of clear correlations between molecular defects and their associated phenotypes [8]. In fact, considering the well-known interindividual differences in expression levels and/or in the functionality of factors responsible for the splicing process and its regulation [31], these dissimilarities could explain the variable clinical manifestations among deficient patients, at least for those individuals carrying splicing mutations.

## 4. Materials and Methods

### 4.1. Materials

Oligonucleotides were purchased from Sigma (St Louis, MO, USA). They were used in sequencing, cloning, and splicing assay (see below) experiments, and their sequence is available on request.

The pTargeT vector was purchased from Promega (Madison, WI, USA). The α-globin-fibronectin hybrid vector (modified pBS-KS) [32] was a kind gift of Dr. Emanuele Buratti (International Centre for Genetic Engineering and Biotechnology, Trieste, Italy).

### 4.2. Coagulation Tests

FV activity and antigen plasma levels were measured as detailed in [33]. Briefly, FV functional assay was based on the prothrombin time; antigen levels were evaluated by using a sandwich enzyme immunoassay, based on a sheep anti-human polyclonal antibody (Affinity Biologicals, Hamilton, ON, Canada). In both tests, FV levels were expressed as percentage of a control plasma mixture (pooling together plasma from 40 normal individuals), set as 100%. Normal ranges for FV:C and antigen FV:Ag levels were 58–140% and 64–139%, respectively. The sensitivity of the functional and immunologic tests was 1% and 0.01%, respectively.

### 4.3. DNA Extraction, PCR Amplifications, and Sequencing

Genomic DNA was extracted from whole blood using an automated DNA extractor (Chemagic Star workstation; Hamilton, ON, Canada) and PCR-amplified under standard conditions, using primer couples designed on the basis of known sequence of the *F5* gene (RefSeq NM_000130). Direct Sanger sequencing of purified PCR products was performed on both strands (using the BigDye Terminator Cycle Sequencing Ready Reaction Kit v1.1; Thermo Fisher Scientific, Waltham, MA, USA) and analyzed on an ABI-3130XL Genetic Analyzer (Thermo Fisher Scientific). The Variant Reporter program (Thermo Fisher Scientific) was used for mutation detection. Sequencing primers were the same as in the amplification reactions, except those used for the 2820-bp-long exon 13, which was sequenced using additional internal primers.

### 4.4. In-Silico Analyses of Splice-Site and Missense Variants

Computer-assisted analysis for splice-site variants was accomplished by using four prediction tools: Human Splicing Finder [34], NetGene2 [35], Splice Site Prediction by Neural Network [36], and ADA [37].

Missense variants were analyzed by using five prediction programs: SIFT [38], PolyPhen2 (two algorithms) [39], MutationTaster [40], and Likelihood Ratio Test (LRT) [41]. These five programs, together with the ADA tool, were run through the Variant Effect Predictor (VEP) online tool maintained in the Ensembl resource (https://www.ensembl.org/info/docs/tools/vep/index.html) (access on 8 January 2019), which takes advantage of the dbNSFP v3.0 databases [42].

Multiple alignments of the FV protein from several vertebrates in the regions surrounding the identified mutations were performed by retrieving FV protein sequences from the UniProt database (https://www.uniprot.org/) (access on 16 December 2018), and by producing the alignment with the CLUSTAL Omega (version 1.2.4) software [43]. Ribbon diagrams of the bovine FVai were produced using the Swiss-Pdb Viewer 4.1 software [44] and the Protein Data Bank 1SDD entry [25].

### 4.5. Mini-Gene Construction

Two *F5* regions (from exon 1 to intron 2, and from intron 18 to intron 21) were PCR amplified from the genomic DNA of the combined heterozygous patient P1, using the Expand 20 KbPLUS PCR System (Roche, Basel, Switzerland) according to the manufacturer’s instructions. PCR products were inserted into the pTargeT vector using the pTargeT Mammalian Expression T-Vector System Kit (Promega). This strategy allowed the production of four plasmids, two wild-type for the above-mentioned *F5* regions, and the other two carrying either the c.158+1G>A or the c.5789G>A mutation. The c.5789-5T>G mutation was instead introduced in the wild-type construct spanning the intron 18-intron 21 region, by the QuickChange Site-Directed Mutagenesis Kit (Stratagene, La Jolla, CA, USA), according to the manufacturer’s instructions. As for the c.6528G>C mutation, we took advantage of the pTargeT-based, wild-type plasmid covering the intron 22-exon 25 region, already described by our group [18]. In this case, the relevant mutation was again introduced by the QuickChange Site-Directed Mutagenesis Kit.

As for the c.5789-11C>A and c.5789-12C>A mutations, for which we had to understand if they were in cis or in trans in patient P3, we PCR amplified a *F5* genomic region (covering exon 20 and its adjacent intronic regions) from the genomic DNA of the patient. PCR products were inserted into the α-globin-fibronectin hybrid vector. This approach revealed that the c.5789-11C>A and c.5789-12C>A mutations are actually in cis, and allowed the production of the corresponding wild-type construct.

All plasmids were isolated by the PureYield Plasmid Midiprep System (Promega) and were checked by sequencing.

### 4.6. Cell Cultures, Transfections, and Splicing Assays

Human cervix carcinoma HeLa cells were cultured in Dulbecco’s modified Eagle’s Medium (DMEM), supplemented with fetal bovine serum (10%), glutamine (1%), and antibiotics (100 IU/mL penicillin, 100 μg/mL streptomycin). Cells were grown at 37 °C in a humidified atmosphere of 5% CO_2_ and 95% air and cultured according to standard procedures. All reagents for cell cultures were from EuroClone (Wetherby, UK).

HeLa cells were seeded in six-well plates at a density of 2.5 × 10^5^/well; 24 h later, transfections were performed using either the wild-type or each of the mutant vector. As a negative control, cells were transfected with the relevant empty plasmid (the pTargeT plasmid, or the α-globin-fibronectin hybrid vector). For each experiment, 4 µg of DNA were transfected using the Fugene reagent (Promega). After 24 h, the medium was removed, cells washed, and RNA was extracted using the Eurozol reagent (EuroClone).

First strand cDNA synthesis, starting from 500 ng of total RNA, was performed using random hexamers and the ImProm-III Reverse Transcriptase (Promega) in a final volume of 20 µL. One µL was used as template for the following PCR amplification, with primers mapping in the upstream and downstream exonic regions of the exon involved in the mutation. Amplified and purified RT-PCR fragments were analyzed by Sanger sequencing, as described above.

All individuals participating to the present study gave their informed consent before blood withdrawal, in accordance with the local Ethics Committees and with the ethical principles of the Helsinki Doctrine.

## Figures and Tables

**Table 1:** Clinical data of the analyzed factor V (FV)-deficient patients.

Patient	Origin	Present Age (Years)	Sex	FV:C (%)	FV:Ag (%)	Main Hemorrhagic Manifestations/Challenges
**P1**	Italy	29	F	3	2	Ecchymoses; Surgery for cysts removal on knee, resulting in post-surgery bleeding and consequent anemia (no treatment used)
**P2**	Italy	52	F	55	53	Two pregnancies, both with premature delivery, and in one case post-delivery bleeding (two days after the event; no treatment); At age four, appendectomy (no complications); At age 32, saphenectomy (no complications); Menorrhagia (no treatment); Implantology surgery (antifibrinolytic treatment);
**P3**	Italy	73	F	5	n.a.	Menorrhagia (no treatment); Tooth extractions performed under prophylactic treatment, but still resulting in bleeding; Two pregnancies (no complications); At age 49, polyp removal from vocal cord (antifibrinolytic treatment); At age 56, polyp removal from uterus (under prophylactic treatment with FFP); At age 59, dilation and curettage procedure (under prophylactic treatment with FFP)
**P4**	Iran	n.a.	F	4	n.a.	n.a.

FV:C, functional FV activity; FV:Ag, antigen FV level (normal ranges: 58% to 140% and 64% to 139% for FV:C and FV:Ag, respectively); F, female; n.a., not available; FFP, fresh-frozen plasma.

**Table 2:** Genetic data of the FV-deficient patients.

Patient	Exon/Intron	Type of Mutation	cDNA Level *	Native Protein	Mature Protein	Status	Gnom AD **	Reference
**P1**	In1 Ex20 Ex24	Splicing Missense or splicing? Missense or splicing?	$\left\{ \begin{array}{l} c.158+1G>A\\ c.5789G>A \end{array}\right.$ c.6528G>C	- p.G1930D p.K2176N	- p.G1902D p.K2148N	Hetero Hetero Hetero	- 4 -	[24] [24] Novel
**P2**	In1 Ex20	Splicing Missense or splicing?	$\left\{ \begin{array}{l} c.158+1G>A\\ c.5789G>A \end{array}\right.$	- p.G1930D	- p.G1902D	Hetero Hetero	- 4	[24] [24]
**P3**	Ex15 In19 In19	Missense Splicing Splicing	c.5090A>G $\left\{ \begin{array}{l} c.5789-11C>A\\ c.5789-12C>A \end{array}\right.$	p.D1697G - -	p.D1669G - -	Hetero Hetero Hetero	- 1 1	Novel Novel Novel
**P4**	In19	Splicing	c.5789-5T>G	-	-	Homo ***	2	Novel

* Numbering starting from the ATG start codon according to RefSeq ENST00000367797.3. ** Number of reported alleles in the GnomAD database (http://gnomad.broadinstitute.org/; if present, the variant was always described in the heterozygous state). *** The mutation is considered to be present in the homozygous state, since the patient’s parents are consanguineous (unknown degree); however, we cannot rule out the possible presence of a large deletion that went undetected during the PCR/direct sequencing strategy used for mutation screening. Curly braces join the two variants characterizing a previously described complex allele [24]. The square bracket joins two adjacent variants that are present in cis in our patient. For missense mutation, the one-letter code annotation was adopted. In, intron; Ex, exon; Hetero, variant found in the heterozygous state; Homo, variant found in the homozygous state.

**Table 3:** In silico predictions of the identified genetic variants.

Variant	Splice-Site Predictions				Missense-Variant Predictions				
	HSF	NetGene2	SSPNN	ADA	SIFT	HumVar	HumDiv	Mutation Taster	LRT
**c.158+1G>A**	60.20 (87.03)	disrupted (0.82)	disrupted (1.00)	0.99	n.p.	n.p.	n.p.	n.p.	n.p.
**c.5789G>A/p.G1902D**	75.80 (79.96)	0.27 (0.53)	0.61 (0.92)	0.98	ND	ND	ND	D	ND
**c.6528G>C/p.K2148N**	65.59 (76.61)	disrupted (0.00)	disrupted (0.68)	0.99	D	D	D	D	D
**p.D1669G**	n.p.	n.p.	n.p.	n.p.	ND	D	D	D	D
**c.5789-11C>A**	61.63 (68.5)	0.30 (0.53)	0.86 (0.92)	4.97E-04	n.p.	n.p.	n.p.	n.p.	n.p.
**c.5789-12C>A**	64.25 (68.5)	0.33 (0.53)	0.77 (0.92)	0.0078	n.p.	n.p.	n.p.	n.p.	n.p.
**c.5789-5T>G**	76.33 (79.96)	disrupted (0.53)	disrupted (0.92)	0.45	n.p.	n.p.	n.p.	n.p.	n.p.

The programs used for splice-site predictions were: Human Splicing Finder (HSF), NetGene2, Splice Site Prediction by Neural Network (SSPNN), and Adaptive Boosting algorithm (ADA). For splice-site prediction using HSF, signals above 65 are considered strong splice sites. If the wild-type score (WT; indicated in parenthesis) is above the threshold, and the score variation between the WT and mutant sequence is higher than 10%, the mutation is considered to break the splice site. For NetGene2 and SSPNN, higher scores imply a higher confidence of true splice sites. As for ADA predictions, scores above 0.7 were used to define a variant as splice altering. The programs used for missense–variant predictions were: SIFT, PolyPhen2 (two algorithms: HumVar and HumDiv), MutationTaster, and the likelihood ratio test (LRT), which were all enclosed in the Variant Effect Predictor (VEP) online tool. D: damaging; ND: not damaging; n.p., not performed.

## Abbreviations

DMEM	Dulbecco’s modified Eagle’s Medium
*F5*	Coagulation factor V gene
FV	Factor V
FVa	Activated factor V
FV:Ag	Factor V antigen level
FVai	Activated protein-C inactivated FVa
FV:C	Factor V coagulant activity
FXa	Activated factor X
GnomAD	Genome Aggregation database
HGMD	Human Gene Mutation Database
PTC	Premature termination codon
RICD	Rare inherited coagulation disorder
WT	Wild type
